# Macro-contextual determinants of cancer screening participation and inequalities: A multilevel analysis of 29 European countries

**DOI:** 10.1016/j.ssmph.2021.100830

**Published:** 2021-05-28

**Authors:** Vladimir Jolidon, Piet Bracke, Claudine Burton-Jeangros

**Affiliations:** a Institute of Sociological Research, University of Geneva, 40 Bd Du Pont-d'Arve, 1205, Genève, Switzerland; b Department of Sociology, Ghent University, Korte Meer 5, 9000, Ghent, Belgium

**Keywords:** Cancer screening participation, Macro-level determinants, Social protection expenditure, Healthcare system, Educational inequalities, Multilevel analysis, European countries

## Abstract

**Background:**

Little attention has been devoted to the role of macro-level determinants in preventive health inequalities, particularly in cancer screening participation. Research has evidenced inequalities in cancer screening uptake yet has mainly focused on the screening programmes’ moderating role at the macro-level. To address this gap, this study examines how welfare provision and healthcare system features modify cancer screening uptake and inequalities across European countries.

**Methods:**

Data from 99 715 (Pap smear) and 54 557 (mammography) women in 29 countries from the European Health Interview Survey (EHIS) 2014 wave and Swiss Health Interview Survey (SHIS) 2012 wave was analysed. We estimated multilevel logistic regression models, including cross-level interactions, to examine whether social protection expenditure in particular policy areas and healthcare system characteristics explained cross-country differences in Pap smear and mammography uptake and inequalities.

**Results:**

Main findings revealed that GP gatekeeping systems were associated with reduced screening uptake likelihood in both Pap smear and mammography, and so were stronger primary care systems in Pap smear, while higher expenditures on old age and survivors were associated with increased mammography uptake. Cross-level interactions showed that in countries with higher expenditures on sickness/healthcare, disability, social exclusion and public health, and a higher number of GPs, educational inequalities in both Pap smear and mammography uptake were smaller, while higher out-of-pocket payments had the opposite effect of increasing inequalities.

**Conclusions:**

Overall, our results show that social protection policies and healthcare system features affect cancer screening participation. We conclude that institutional and policy arrangements interact with individuals’ (educational) resources and, through the (re)distribution of valued goods and resources at the macro level, these arrangements may contribute to enhancing preventive healthcare use and mitigating screening uptake inequalities.

## Highlights

• Welfare provision and healthcare system features affect Pap smear and mammography uptake.
• Social spending and healthcare features moderate inequalities in cancer screening uptake.
• Policymakers should consider macro-level factors for cancer screening strategies and programmes.
• Policies and institutions shape social determinants of preventive healthcare use.

## 1 Introduction

Increasing attention has been paid to macro-contextual and institutional determinants of health and health inequalities in social science and epidemiology (Beckfield & Krieger, 2009; Brennenstuhl et al., 2012; Muntaner et al., 2011). This ‘institutional turn’ in cross-national studies has drawn attention to macro-level determinants and how these interact with socioeconomic differences in health at the individual level (Beckfield et al., 2015). From this standpoint, social policy contexts and healthcare characteristics shape not only the distribution of resources in a population, but also to what extent these resources are important for individuals' health across different institutional settings.

In spite of this institutional turn, little attention was dedicated to the macro-contextual determinants of *preventive* healthcare (Missinne et al., 2014; Willems et al., 2020). We should note that qualitative historical research has examined how the interactions between healthcare and public health sectors affect preventive health policies, with an institutionalist approach in comparative policy analysis (Trein, 2018). Nevertheless, research on preventive healthcare use and inequalities was strongly influenced by social epidemiology and primarily concerned with the identification of individual risk factors and how health behaviours relate to unequal socioeconomic conditions (Frohlich & Abel, 2014). Similarly, in cancer screening participation, research has mainly focused on the role of psychosocial determinants, emphasising individual behaviour, and has overlooked context and system-level factors (Priaulx et al., 2020; Sarma, 2015).

Socioeconomic inequalities in cervical and breast cancer screening tests were evidenced to persist over time and across European countries (Damiani et al., 2015; Jolidon et al., 2020; Sabates & Feinstein, 2006; Willems & Bracke, 2018; Wubker, 2014). Nevertheless, the only country-level factors research has addressed are the cancer screening programmes (Palencia et al., 2010; Walsh et al., 2011; Willems & Bracke, 2018) and macrolevel gender inequality (Willems et al., 2020). To address this gap, the present paper scrutinises the moderating effect of further macro-contextual and institutional determinants of cross-national variation in cancer screening uptake, particularly welfare state provision and healthcare system characteristics, and how these affect inequalities in screening participation.

## 2 Background

The institutional approach to health and health inequalities stresses the role of policy and institutional arrangements which structure social inequalities and (re)distribute social determinants of population health (Beckfield et al., 2015). In this sense, welfare and healthcare system arrangements have ‘institutional effects’ (Beckfield et al., 2015) which might directly or indirectly affect cancer screening participation, and thus explain cross-country differences in screening uptake and inequalities. Social inequalities in health persist due to the uneven distribution of resources, such as status, knowledge, money, power, and social networks, which people can deploy to avoid disease and adopt protective strategies (Phelan et al., 2010). Among these, education is a key resource which involves knowledge and skills that individuals can translate into healthier lifestyles, and it was shown to enhance participation in healthcare services (Terraneo, 2015) and cancer screening (Willems & Bracke, 2018). The higher educated are better equipped to navigate the complexities of the healthcare system while the lower educated face greater power asymmetries defined by the healthcare sociocultural and organisational environment and, thus, may experience more informational and cultural barriers to healthcare access. Moreover, the higher educated were shown to favour prevention-focused health decisions, while the lower educated may sometimes lack of incentive to undertake asymptomatic screening of which they may not see the immediate benefits (Sabates & Feinstein, 2006; Kim et al., 2008). Education, the social inequality measure chosen in this paper, was shown to be a robust measure of socioeconomic position in comparative European analyses (Mackenbach et al., 2008) and to capture the social distribution of a large range of health determinants, as it relates to material, psychological and social resources (Ross & Wu, 1995).

At the macro (country) level, welfare provision through social policies was shown to affect population health and health inequalities (Bergqvist et al., 2013; Brennenstuhl et al., 2012). Using social protection expenditure as an indirect measure of welfare state generosity, studies showed that higher social spending was associated with better health and lower health inequalities (Dahl & van der Wel, 2013). Indeed, collectively provided ‘welfare resources’ contribute to compensating for market and family failures and are crucial to population health (Lundberg et al., 2008). Since social policies provide individuals access to resources (in cash and in kind) which strengthen their agency, expand their capacity to cope with stressful events and enhance their social integration and participation (Dahl & van der Wel, 2013; Visser et al., 2018), we may expect these to play a role in cancer screening participation. In market societies, financial resources can reduce direct and indirect barriers to healthcare access (Israel, 2016) and be converted into health enhancing resources such as participation in preventive healthcare. Thus, as social policies address the unequal distribution of resources and positively affect the social determinants of *preventive* health, we expect these to increase cancer screening uptake, and, since these might particularly benefit the more disadvantaged who have less resources to count on, we also expect social policies to reduce screening uptake inequalities. This welfare resource perspective thus supports the expenditure approach adopted by the present study as an analytical strategy**.** Regime approaches were criticised for relying on typologies which group welfare states along shared characteristics but fail to reveal the mechanisms linking specific macro-level determinants and individual-level outcomes (Bergqvist et al., 2013). Hence, in line with an expenditure approach, we disaggregate social spending and examine the effect of specific policy areas in cancer screening uptake and inequalities (de Breij et al., 2020).

Healthcare system characteristics affect access to healthcare resources and availability and deserve particular attention in relation to cancer screening participation. Since healthcare systems remained relatively absent from major welfare state theories, Bambra (2005) introduced the healthcare decommodification concept to account for the degree to which the provision of healthcare is independent from the market, and the extent to which an individual's access is dependent on his or her market position (Bambra, 2005). This concept highlights the social role of the ‘health-care state’ and its characteristics, which are responsible for the decommodification of healthcare, and are key dimensions of an institutional and comparative perspective (Reibling, 2010). The healthcare system mode of financing may impact healthcare access (Carrin et al., 2008), through its degree of (de)commodification. Namely, an increased public health expenditure (PHE) may reduce the importance of private actors in the healthcare system and distribute health cost sharing more equally among the population (Kim et al., 2017). In addition to this public-private mix perspective, we take into account healthcare characteristics and institutional regulations which determine the conditions of access to care and the services which can potentially be received, such as the gatekeeping system (GP referral), financial dis-incentives (out-of-pocket payments) and service provision intensity (number of doctors). These features account for access to care from the recipients' perspective**.** They decommodify healthcare because they make individuals' access less dependent upon the market and their market position (Reibling, 2010), and were shown to be associated with healthcare access and cancer screening uptake (Jusot et al., 2012; Reibling & Wendt, 2011). We also stress the potential role of the primary care system in cancer screening participation since it was evidenced to enhance access and reduce inequalities in healthcare access (Detollenaere et al., 2017; Kringos, 2012; Kringos et al., 2013).

This study focuses on cervical and breast cancer screening, since most European countries have implemented Pap smear and mammography population-based screening based on EU recommendations to systematically screen all women within specific age intervals (Council of the European Union, 2003). Thus, we focus on these two screening tests which are well-established across countries. We have not included colorectal screening since population-based screening recommendations are more recent and their implementation has higher cross-country variation, and thus its uptake prevalence is much lower than Pap smear and mammography. More gender unequal contexts were shown to negatively affect women's Pap smear and mammography uptake (Willems et al., 2020). That is, policy and institutional contexts shape the gendered distribution of resources and agency which may constrain women's ability to prioritise preventive healthcare and engage in cancer screening. Therefore, it is of interest to scrutinise how welfare provisions and healthcare arrangements may support women in their cancer screening participation and reduce screening inequalities.

We should note that most European countries have implemented organised screening programmes for breast and cervical cancer screening, yet some programmes only achieve a partial coverage of the target population and others are only implemented regionally or at a ‘pilot phase’ (IARC, 2017). Those programmes coexist with ‘opportunistic screening’, namely, individual screening through primary care, GPs and specialists, which relies on individual initiative or the doctor's recommendation to screen.

To our knowledge, no study has addressed the role of both specific welfare state policies and healthcare characteristics in cancer screening uptake and inequalities. Additionally, no study has examined the macro-contextual determinants of cancer screening inequalities using multilevel models with random slopes specification for individual (lower-level) variables in cross-level interactions. As pointed out by Heisig and Schaeffer (2019), numerous multilevel studies have examined cross-level interactions with random intercept-only models. These models are more likely to report statistically significant cross-level interaction effects since such model ‘misspecification’ leads to anti-conservative inference for both the lower-level interaction term and main effect component.

In the present study, we address the following questions and hypotheses:

1. How are welfare social policies and healthcare system characteristics related to cancer screening participation? We hypothesise that more generous welfare provision and decommodifying healthcare system arrangements are associated with higher cancer screening participation.
2. Do welfare social policies and healthcare system characteristics modify the association between education and cancer screening participation? We hypothesise that higher social spending and decommodifying healthcare arrangements benefit the lower educated more than higher educated groups and thus are associated with smaller educational inequalities in cancer screening.

This study adds to the comparative literature on cancer screening participation by examining the role of additional macro-level determinants in modifying cancer screening inequalities and analysing specific policy areas, rather than solely attending to total expenditure measures or aggregated indices, and healthcare system characteristics.

## 3 Methods

### 3.1 Data

**Fig. 1:**
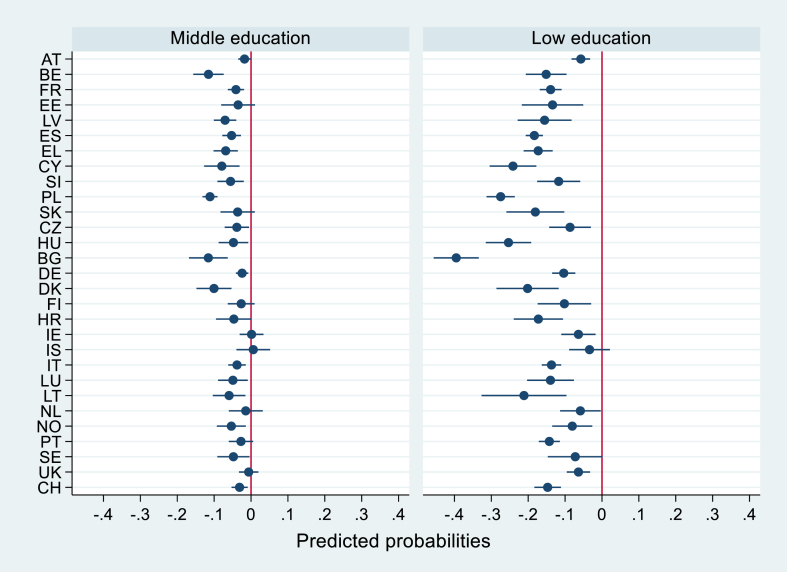
Country-specific middle and low education marginal effects on Pap smear uptake, with 95% confidence intervals Note: Marginal effects are the differences between the predicted probabilities (PPs) of middle and low educated groups, and their reference category (high education). PPs are based on country-specific logistic regression models adjusted for age, cohabitation status, and visit to a GP in the past 12 months.

**Fig. 2:**
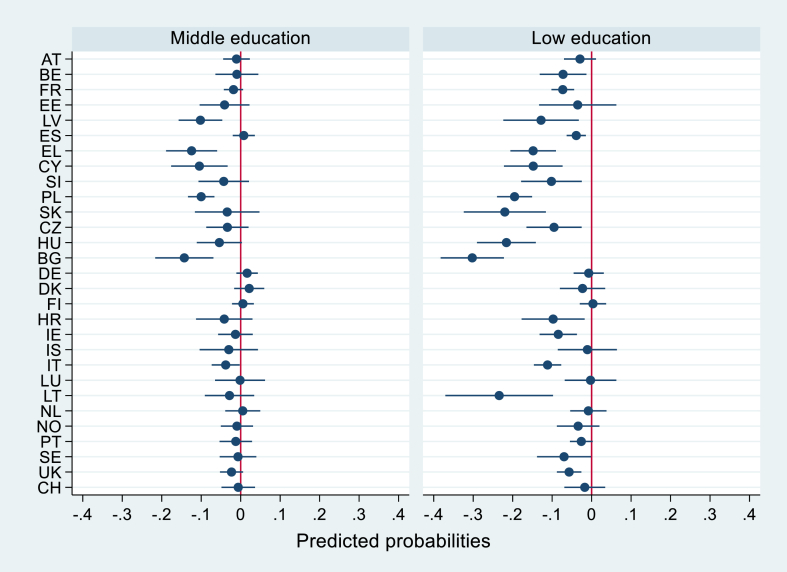
Country-specific middle and low education marginal effects on mammography uptake, with 95% confidence intervals Note: Marginal effects are the differences between the predicted probabilities (PPs) of middle and low educated groups, and their reference category (high education). PPs are based on country-specific logistic regression models adjusted for age, cohabitation status, and visit to a GP in the past 12 months.

We analysed data from the 2014 wave of the European Health Interview Survey (Eurostat, 2014). The EHIS consists of four modules on healthcare use, health status, health determinants and socio-economic background and focuses on population aged 15 years old and older, and living in private households (Eurostat, 2018). Data collection took place between 2013 and 2015 in 30 European countries, including Norway and Iceland. We excluded Romania and Malta from our analysis since the former was an outlier (see Fig. 1, Fig. 2, supplementary materials) and the latter did not have ‘high education’ cases in the education variable. We added data for Switzerland from the 2012 Swiss Health Interview Survey (Swiss Federal Office of Public Health, 2012) since the variables analysed in our study matched between the two survey datasets, except for country of birth as detailed in supplementary materials (Table S.1). Hence, our final sample comprised 29 countries. Sample sizes were restricted to the recommended screening age intervals for each cancer screening test, according to the European screening guidelines (IARC, 2017): women 25–64 years old for Pap smear and 50–69 years old for mammography. To ensure that the outcomes occurred after the respondents had completed their education, students (n_Pap smear_ = 1,624, 1.7%; n_mammography_ = 420, 0.8%) were not included in the study sample. We excluded cases with missing information in predictors (n_Pap smear_ = 2,821, 2.6%; n_mammography_ = 1,598, 2.8%) and outcomes (n_Pap smear_ = 1,219, 1.1%; n_mammography_ = 394, 0.7%) and the final samples consisted of 99 715 women for Pap smear and 54 557 women for mammography. Descriptive statistics of individual and macro-level characteristics for the 29 countries included in this study were reported in Tables S.2, S.3a and S.3b (supplementary materials).

**Table 1:** Multilevel models with associations of education with Pap smear and mammography uptake.

	Pap Smear (n = 99 715)	Mammography (n = 54 557)
	OR (SE)	OR (SE)
**Model 0 (intercept-only)**		
MOR	1.568	2.001
VPC	0.063	0.140
**Model 1a (education crude)**		
Education (ref: high)		
Middle education	0.734*** (0.014)	0.869*** (0.025)
Low education	0.399*** (0.009)	0.626*** (0.020)
MOR	1.568	2.047
VPC	0.063	0.146
**Model 1b (education adjusted)**		
Education (ref: high)		
Middle education	0.802*** (0.016)	0.868*** (0.026)
Low education	0.511*** (0.012)	0.646*** (0.022)
Screening programme (ref: organised)		
Partial programme	1.401 (0.312)	0.682 (0.236)
No programme	1.312 (0.267)	0.482 (0.207)
GDP per capita	2.107 (1.203)	5.951* (4.825)
MOR	1.549	1.830
VPC	0.060	0.109

*p value ≤ 0.05, **p value ≤ 0.01, ***p value ≤ 0.001.

MOR = median odds ratio, VPC = variance partitioning coefficient.

Note: Model 1b was adjusted for age, cohabitation status, self-rated health, area of residence, work status, country of birth, visit to a GP in the past 12 months.

### 3.2 Measures

#### 3.2.1 Dependent variables

Respondents were asked when they last had a cervical smear test (Pap smear) and a mammography (breast X-ray): ‘within the past 12 months, 1 to less than 2 years ago, 2 to less than 3 years ago, more than 3 years ago, or never’. Following the European guidelines on recommended screening intervals for each cancer screening tests, we recoded these answer categories into two binary variables: ‘had a mammography in the past 2 years’ and ‘had a Pap smear in the past 3 years’, (0) no, (1) yes.

#### 3.2.2 Individual-level independent variable

Educational attainment was measured as the highest level of education completed, based on the ISCED-2011 classification from 0 (pre-primary education) to 8 (tertiary education, doctoral level or equivalent). We recoded this variable into three categories for a better consistency in the education variable operationalisation across all countries: (0) low education (ISCED 0, 1 and 2), (1) middle education (ISCED 3 and 4), and (2) high education (ISCED 5, 6, 7 and 8).

#### 3.2.3 Country-level independent variables

Social protection expenditure was measured as a percentage of the GDP in eight social policy areas: sickness/healthcare, disability, old age, survivors, family/children, unemployment, housing and social exclusion, as provided by the Eurostat (definitions in Table S.4, supplementary materials). These expenditures comprise all interventions from public or private bodies, in cash or in kind, intended to relieve households and individuals of the burden of a defined set of risks or needs (Eurostat, 2019). We examined each policy area in separate models. For healthcare characteristics, we analysed separately: private out-of-pocket payments (OOPP) as a percentage of the total health expenditure (THE), public health expenditure (PHE) as a percentage of the GDP, number of GPs and gynaecologists per 100 000 population, which were obtained from the Eurostat, GP gatekeeping system (GP referral), obtained from the OECD (2012) and European Observatory on Health Systems and Policies (2012). We also analysed an indicator of the primary care (PC) system “overall structure strength” provided by Schafer et al. (2015) which captures dimensions of the PC system governance, economic conditions and workforce development (Kringos, 2012). The PC strength indicator was not available for Croatia hence analysis on this indicator excluded Croatia data. Due to their cross-national differences in structure and organisation, it is difficult to compare PC systems across countries. Therefore, we also provided analyses of a proxy for PC expenditure (PCE) in supplementary materials which we created by aggregating expenditures on ‘general outpatient and home-based curative care’ and ‘outpatient and home-based long-term care’ provided by the Eurostat (European Commission, 2014). Since a strong gatekeeping system combines both GP referral and GP paid by capitation (Reibling, 2010), we analysed one model with a categorical variable coded as (0) no GP referral, (1) GP referral, and another model with the variable (0) no GP referral, (1) GP referral and GP paid by capitation. The cancer screening strategies of countries were classified as (0) ‘organised’, if an organised population-based programme was implemented nationwide, (1) ‘partial’ if the programme was not fully implemented (pilot/rollout ongoing/regional programmes) and (2) ‘no programme’, if no programme was implemented, based on Willems et al. (2020) categorisation and IARC information (IARC, 2017). A country's cancer screening strategy was considered two and three years before the time of a country's survey for mammography and Pap smear respectively, taking into account screening interval recommendations. All other macro-contextual variables were measured in 2013.

#### 3.2.4 Confounders

We controlled models for age (5-year age ranges), cohabitation status (living in couple, living alone, other), self-rated health (good, poor), area of residence (urban, rural), work status (employed, non-employed), country of birth (native, born abroad), and last visit to a general practitioner (more, less than 12 months ago). At the macro level, all models were controlled for GDP per capita and models which examined unemployment expenditure were also adjusted for national unemployment rates, since these characteristics may affect the governments’ general ability and need to invest in social protection and healthcare. GDP per capita were obtained from the World Bank and logged to reduce the influence of outliers, and unemployment rates from the Eurostat.

### 3.3 Statistical analyses

We used multilevel logistic models since we had a data structure with individuals (level 1) nested in countries (level 2) and binary outcome variables. The multilevel approach aims to address the interdependencies among observations belonging to the same cluster. The analyses were conducted in MLwiN 3.04 using 2nd order PQL method with starting values based on 1st order MQL (Rasbash et al., 2019). All models were performed for both Pap smear and mammography outcome variables and all continuous macro-contextual variables were grand mean centred across countries, for a better interpretation of the coefficients in the cross-level interaction models.

First, an intercept-only model with a random intercept for country was built to examine whether there were cross-country differences in cancer screening (model 0), using a Wald test. The random intercept-only logistic model can be expressed by the following regression equation:

Log (Y)_ij_ = β_0j_ + u_0j_

Where u0_j_ is the between-country variance. In model 1a, the crude association of education and cancer screening uptake was modelled, and in model 1b this association was adjusted for the confounders.

Second, we built separate models for each macro-level variable and adjusted for the confounders (models 2) to address our first research question: ‘How are welfare resources and healthcare system characteristics related to cancer screening participation?’ To estimate the relative importance of macro-contextual factors, we calculated the variance partitioning coefficients (VPC) and median odds ratios (MOR) – both measures of higher level residual heterogeneity. The VPC quantifies the proportion of country level variance over the total model variance and is calculated with the ‘latent variable method’ (Merlo et al., 2006). We also reported the MOR for better comparison since the VPC can vary depending on the outcome prevalence. The MOR is “the median value of the odds ratio between the country at highest risk and the country at lowest risk when randomly picking out two countries” (Merlo et al., 2006) and a higher MOR indicates larger cross-country differences.

Third, we added random coefficients for education at country level to account for differences in the effect of education on cancer screening in different countries. Random coefficients for “middle” and “low” education were added to the equation (their base level being ‘high’ education):

Log (Y)_ij_ = β_0j_ + β1_j_(middle education X1)_ij_ + β2_j_(low education X2) _ij_ + u_0j_ + u1_j_X_ij_ + u2_j_X_ij_

We used a Wald test to examine cross-country differences in the effect of education on cancer screening uptake. Additionally, we plotted the middle and low education marginal effects on cancer screening participation for each country in Fig. 1, Fig. 2 to further portray the education-based inequalities in cancer screening. Marginal effects were calculated as the differences in the predicted probabilities between the middle and low educated groups and their reference category (high education). Predicted probabilities (PPs) for education levels were calculated based on adjusted odds ratios (ORs) resulting from country-specific logistic regression analyses. PPs are preferred to report differences in logistic regression coefficients because they do not require the assumption that the error variance is identical across countries. By-country logistic regressions, marginal effects and plots were performed on Stata 16.

We then added cross-level interactions between education and the macro-level variables to address our second research question: ‘Do welfare resources and healthcare system characteristics modify the association between education and cancer screening participation?’

Adding a cross-level interaction leads to the following regression equation:

Log (Y)_ij_ = β_0j_ + β1_j_(X1)_ij_ + β2_j_(X2)_ij_ + β3(macro-level X3)_j_ + β4(X1)_ij_ ∗ (macro level X3)_j_ + β5(X2)_ij_ ∗ (macro level X3)_j_ + u_0j_ + u1_j_X_ij_ + u2_j_X_ij_

As Heisig and Schaeffer (2019) pointed out, the level-1 random coefficient specification takes into account cluster-induced heteroskedasticity and cluster-correlated errors (which are the main motivations for multilevel modelling). Failure to do so may produce overly optimistic statistical inference for the cross-level interaction term and the (main effect) coefficient of the lower-level variable involved in the interaction.

For significant cross-level interactions, we calculated and plotted predicted probabilities of cancer screening by education levels and contextual variables based on the coefficients obtained in the regression analyses. This approach allowed depicting absolute inequalities in cancer screening by combining interaction coefficients with their main effects.

## 4 Results

The intercept-only model (model 0) reveals significant cross-country differences in cancer screening (Pap smear: Wald = 13.73, p < 0.001, mammography: Wald = 13.88, p < 0.001). The VPC is 6.3% and 14.0%, for Pap smear and mammography respectively, and represents the total variance in cancer screening uptake which can be attributed to country-level characteristics (Table 1). The MOR is 1.568 and 2.001 indicating that an individual from a country with high screening uptake had 1.568 (Pap smear) and 2.001 (mammography) times the odds of screening (in median) compared to an individual from a country with low screening uptake, which suggests that country-level factors have less influence on Pap smear than on mammography uptake.

Model 1a (Table 1) shows an education gradient in which middle and low education levels are associated with lower screening uptake compared to the higher education level, in both Pap smear and mammography. In model 1b adjusted for all individual (level 1) variables and macro-level (level 2) screening programme and GDP per capita variables, estimators reduced but the education gradient remained. Random coefficients for education levels were added to the models. The effect of middle and low education significantly varied across countries for Pap smear (middle: Wald = 7.65, p = 0.022, low: Wald = 10.49, p = 0.005) while only the effect of low education varied across countries for mammography (middle: Wald = 4.69, p = 0.096, low: Wald = 8.03, p = 0.018). Fig. 1, Fig. 2 depict the marginal effects of middle and low education on cancer screening participation in each country, which were calculated with separate by-country regression models. The figures evidence the existing cross-country differences in the association of cancer screening with education levels.

Models 2 (Table 2) examine each macro-level variable's effect on cancer screening uptake separately. GP referral and capitation system is significantly associated with reduced women's screening uptake likelihood compared to not having a GP referral and capitation system, in both Pap smear and mammography. In Pap smear, a stronger PC system and higher OOPP show a similar effect of reducing uptake likelihood. Although the latter estimator is significant at a 10% level, the model VPC reduces to 5.5%. In mammography, higher old age and survivors expenditures is associated with increased screening uptake likelihood, and so is unemployment expenditure at a 10% significance level (the model VPC reduced to 8.5%). Of these macro-level determinants, the model with GP referral and capitation explains most of the between-country variance in Pap smear (VPC: 4.5%) and the one with old age expenditure in mammography (VPC: 8.2%).

**Fig. 3:**
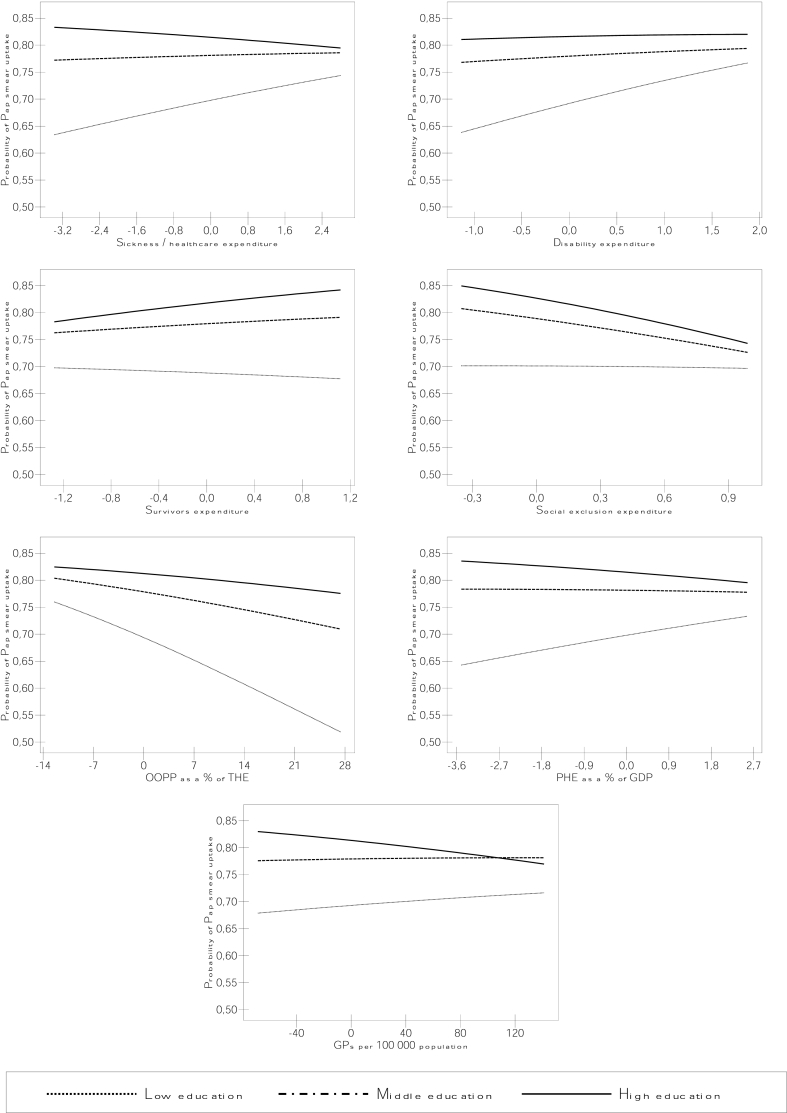
Predicted probabilities of Pap smear uptake by country-level determinants and education levels. Note: OOPP = out-of-pocket payments; THE = total health expenditure; PHE = public health expenditure; GP = general practitioner.

**Fig. 4:**
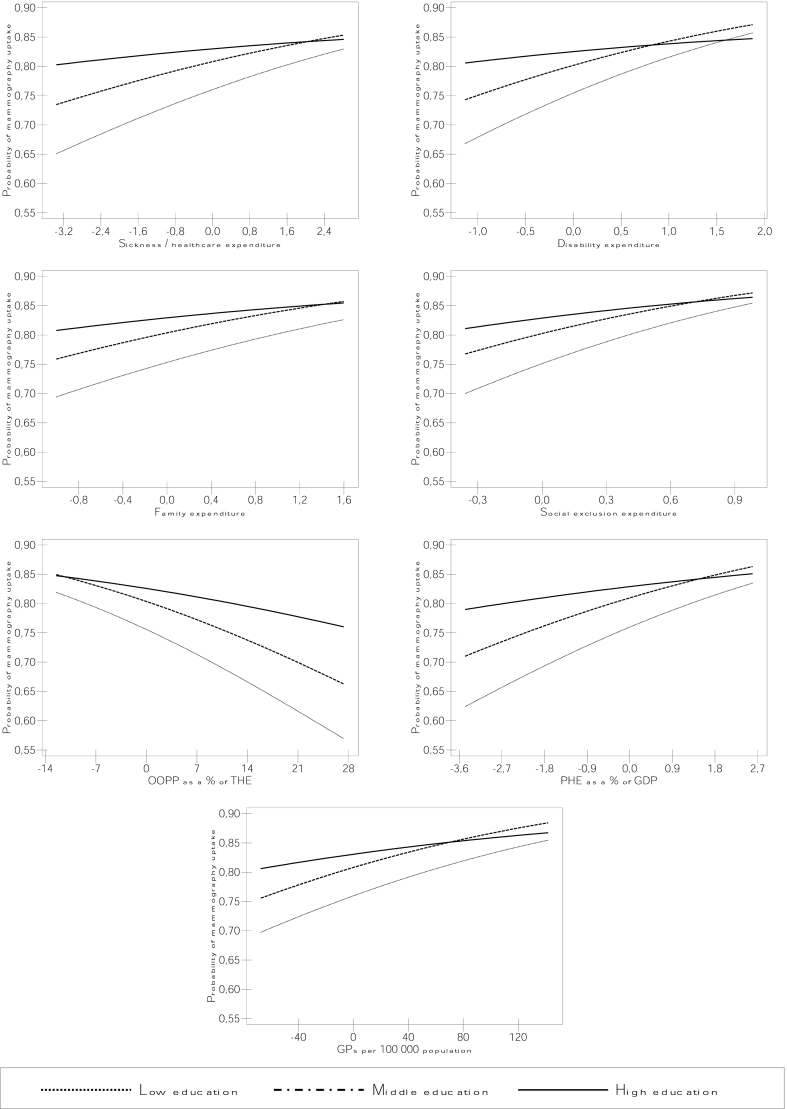
Predicted probabilities of mammography uptake by country-level determinants and education levels. Note: OOPP = out-of-pocket payments; THE = total health expenditure; PHE = public health expenditure; GP = general practitioner.

Table 3 presents models with cross-level interactions between education levels and macro-level factors (model 3). The macro-level variable ORs (first two columns) are the ‘main effects’, which represent the effect of the macro-level variable on cancer screening uptake in the higher education group (the education variable reference category). The interaction terms reveal whether educational differences in macro-level determinants of cancer screening are observed. In countries with higher sickness/healthcare expenditure and public health expenditure (PHE), the middle and lower educated groups have a higher likelihood of both Pap smear and mammography uptake compared to the higher educated group, as significant interaction terms revealed. A similar effect is found with disability and social exclusion expenditure for the lower educated group, and with the number of GPs for the middle educated group, as well as with stronger PC systems for all education groups, although the latter is only significant at a 10% level. The analysis of primary care expenditure (PCE) provides support to this PC strength effect as higher PCE is significantly associated with smaller screening uptake differences between education groups (Table S.6). In mammography, higher disability, family and social exclusion expenditures are also associated with increased uptake likelihood for the middle educated compared to the higher educated group, and a higher number of GPs has a similar effect for the lower educated group in Pap smear. As OOPP increase, the screening uptake likelihood of the lower educated reduces compared to the higher educated group in both screening tests, and a similar effect is found for the middle educated in mammography. This effect is also observed with survivors expenditure for the lower educated group in Pap smear. As shown by the converging lines of predicted probabilities plots in Fig. 3, Fig. 4, as sickness/healthcare, disability and exclusion expenditures, PHE and the number of GPs increase, educational differences in both Pap smear and mammography uptake reduce. A similar pattern is observed with stronger PC systems and higher PCE (Fig. 3, Fig. 4). With the increase of these same macro-level factors, the screening uptake predicted probabilities of the lower and middle educated groups increase in mammography, while in Pap smear such positive slopes are only observed for the lower educated group in sickness and disability expenditure, PHE, PCE and the number of GPs.

**Table 2:** Multilevel models with associations of macro-level variables with Pap smear and mammography uptake.

	Model 2					
	Pap Smear (n = 99715)			Mammography (n = 54 557)		
	OR (SE)	MOR	VPC	OR (SE)	MOR	VPC
**Social protection expenditure**						
Sickness/healthcare	1.006 (0.063)	1.548	0.060	1.139 (0.094)	1.785	0.101
Disability	1.072 (0.151)	1.546	0.060	1.339 (0.242)	1.786	0.101
Old age	1.035 (0.047)	1.543	0.059	1.199** (0.067)	1.675	0.082
Survivors	1.197 (0.187)	1.533	0.058	1.464* (0.239)	1.738	0.093
Families/children	1.194 (0.158)	1.527	0.056	1.26 (0.237)	1.806	0.105
Unemployment	1.086 (0.147)	1.545	0.059	1.331 (0.227)	1.695	0.085
Housing	1.016 (0.307)	1.547	0.060	1.331 (0.565)	1.820	0.107
Social exclusion	0.667 (0.186)	1.524	0.056	1.766 (0.668)	1.792	0.102
***Health care system characteristic***						
OOPP % THE	0.980 (0.012)	1.519	0.055	0.973 (0.014)	1.769	0.098
PHE % GDP	0.984 (0.062)	1.548	0.060	1.168 (0.095)	1.766	0.097
PC strength	0.313* (0.168)	1.501	0.052	3.197 (2.484)	1.804	0.104
Nr of GPs	0.999 (0.002)	1.547	0.060	1.004 (0.003)	1.781	0.100
Nr of gynaecologists	1.032 (0.020)	1.521	0.055	0.959 (0.023)	1.777	0.099
GP referral (ref: no referral)	0.532** (0.112)	1.465	0.046	1.023 (0.286)	1.831	0.109
GP referral & capitation (ref: no referral)	0.567** (0.102)	1.457	0.045	0.575* (0.144)	1.749	0.095

*p value ≤ 0.05, **p value ≤ 0.01, ***p value ≤ 0.001.

MOR = median odds ratio, VPC = variance partitioning coefficient, OOPP = out-of-pocket payments, THE = total health expenditure, PHE = public health expenditure, PC = primary care.

Note: Random intercept models adjusted for education, age, cohabitation status, self-rated health, area of residence, work status, country of birth, visit to a GP in the past 12 months, cancer screening programme and GDP per capita. The unemployment expenditure model was also adjusted for national unemployment rates.

**Table 3:** Cross-level interactions between education and macro-level variables in their effect on cancer screening participation.

	Model 3			
	Macro-level variable		Education * Macro-level variable	
	Pap Smear	Mammography	Pap Smear	Mammography
	OR (SE)	OR (SE)	OR (SE)	OR (SE)
***Social protection expenditure***				
Sickness * middle edu.	0.959 (0.065)	1.054 (0.089)	1.058** (0.021)	1.080*** (0.019)
Sickness * low edu.			1.141*** (0.039)	1.123** (0.041)
Disability * middle edu.	1.022 (0.158)	1.116 (0.211)	1.029 (0.056)	1.224*** (0.073)
Disability * low edu.			1.215* (0.114)	1.331** (0.125)
Old age * middle edu.	1.043 (0.055)	1.221*** (0.065)	0.986 (0.020)	0.979 (0.024)
Old age * low edu.			0.971 (0.035)	0.973 (0.037)
Survivors * middle edu.	1.19 (0.199)	1.488* (0.244)	0.905 (0.046)	0.991 (0.062)
Survivors * low edu.			0.809* (0.076)	0.96 (0.099)
Familiy * middle edu.	1.411** (0.182)	1.157 (0.220)	1.011 (0.049)	1.136* (0.058)
Familiy * low edu.			1.098 (0.099)	1.186 (0.107)
Unemployment * middle edu.	1.025 (0.149)	1.274 (0.246)	1.026 (0.046)	1.088 (0.058)
Unemployment * low edu.			1.043 (0.088)	1.044 (0.094)
Housing * middle edu.	1.055 (0.354)	1.205 (0.512)	1.192 (0.125)	1.19 (0.140)
Housing * low edu.			1.464 (0.292)	1.344 (0.273)
Social exclusion * middle edu.	0.608 (0.186)	1.414 (0.548)	1.176 (0.127)	1.303* (0.169)
Social exclusion * low edu.			1.642** (0.303)	1.528* (0.310)
***Health care system characteristic***				
OOPP % THE * middle edu.	0.993 (0.013)	0.986 (0.015)	0.994 (0.004)	0.986*** (0.004)
OOPP % THE * low edu.			0.979*** (0.006)	0.981** (0.007)
PHE % GDP * middle edu.	0.953 (0.064)	1.071 (0.098)	1.042* (0.022)	1.093*** (0.018)
PHE % GDP * low edu.			1.129*** (0.039)	1.127** (0.041)
PC strength * middle edu.	0.246* (0.147)	1.834 (1.542)	1.494 (0.350)	1.733 (0.488)
PC strength * low edu.			2.264 (0.957)	2.111 (0.960)
Nr of GPs * middle edu.	0.998 (0.002)	1.003 (0.003)	1.002** (0.001)	1.002** (0.001)
Nr of GPs * low edu.			1.003* (0.001)	1.002 (0.001)
Nr of gynaecologists * middle edu.	1.043 (0.023)	0.961 (0.023)	0.991 (0.007)	0.991 (0.008)
Nr of gynaecologists * low edu.			0.975 (0.013)	0.994 (0.014)
GP referral * middle edu.	0.591* (0.132)	1.016 (0.288)	1.052 (0.083)	0.969 (0.089)
GP referral * low edu.			1.065 (0.155)	0.91 (0.135)
GP referral & capitation * middle edu.	0.586** (0.119)	0.614 (0.157)	1.004 (0.078)	0.914 (0.080)
GP referral & capitation * low edu.			0.985 (0.140)	0.848 (0.120)

*p value ≤ 0.05, **p value ≤ 0.01, ***p value ≤ 0.001.

Pap smear n = 99 715, Mammography n = 54 557.

OOPP = out-of-pocket payments, THE = total health expenditure, PHE = public health expenditure, PC = primary care.

Note: Random coefficients where included for education. Models were adjusted for age, cohabitation status, self-rated health, area of residence, work status, country of birth, visit to a GP in the past 12 months, cancer screening programme and GDP per capita. The unemployment expenditure model was also adjusted for national unemployment rates.

## 5 Discussion

This study examined the role of macro-level determinants in Pap smear and mammography uptake and how these moderate education-based inequalities in these two screening tests. Little research has focused on country-level determinants of cancer screening inequalities, except for the effect of cancer screening programmes (Palencia et al., 2010; Walsh et al., 2011; Willems & Bracke, 2018). We examined specific social policy areas, with a social protection expenditure approach, and healthcare system characteristics as determinants of cancer screening uptake and inequalities.

Our first hypothesis that more generous social policies and decommodifying healthcare system arrangements were associated with higher cancer screening uptake was partially supported by our results since only some social policy areas and healthcare system features were associated with screening uptake levels. In Pap smear, countries with higher OOPP had a lower screening uptake, which is in line with our hypothesis. GP gatekeeping and stronger primary care (PC) systems, both decommodifying features, were associated with reduced screening uptake in opposition to our hypothesis. This could be explained by the prevailing overscreening in Pap smear uptake (De Prez et al., 2020), particularly in opportunistic screening contexts, which is often due to gynaecologists performing Pap smear as part of routine check-ups on a yearly basis. In this regard, gatekeeping and stronger PC systems may act as regulatory mechanisms, controlling and containing the specialist visits and possibly limiting unnecessary screening. Although gatekeeping systems with capitation payments can be designed to improve access to preventive care, research showed that such systems may also reduce service use in general, particularly since stronger gatekeeping systems have healthcare resources limited by central budgets (Jusot et al., 2012). Stronger PC systems are usually characterised by GP gatekeeping systems and tighter control over healthcare resources (Kringos, 2012). In mammography, higher old age and survivors expenditures were associated with higher screening uptake. The first two factors are relevant to the sample age range and may contribute to fostering mammography participation, particularly among older women who were found to screen less than their younger peers (Wuebker, 2012). Social protection spending in relevant policy areas provide the resources which may help tackle the restricted agency and competing choices faced by women. This in turn may enhance their participation in cancer screening, especially in more gender unequal contexts (Willems et al., 2020). As research showed, women's health is sensitive to public welfare and improved by higher social welfare levels (Raphael & Bryant, 2004). In policy contexts which leverage their autonomy and agency, they are more likely to use health services (Osamor & Grady, 2016) and prioritise preventive health.

Our second hypothesis that more generous welfare provisions and decommodifying healthcare system arrangements were associated with reduced cancer screening inequalities found relevant support in our results. Countries with stronger PC systems had smaller educational inequalities in both Pap smear and mammography uptake. Although this effect was only significant at a 10% level, it was supported by the finding that higher primary care expenditure (PCE) was significantly associated with smaller screening inequalities (Table S.6). Disadvantaged groups may benefit more from stronger PC systems than the more advantaged. These systems have usually committed to universal access to healthcare, including lowering co-payments, and GPs occupy a central role as the system main entry point, with policies aimed at enhancing GP access as well as the GP role in first contact care, medical advocacy and patient care coordination (Kringos, 2012). Hence, stronger PC systems are associated with better person-focus care: healthcare providers are more likely to be involved in a larger range of health problems at different stages of the patient's lives, and patients have a better perception of the healthcare system (Kringos, 2012; Kringos et al., 2013; Schafer et al., 2015). Our results also confirmed the GP's role in prevention, as countries with higher number of GPs had smaller screening inequalities between education levels. GPs may play an important role considering that the more disadvantaged utilise specialist care less than their more advantaged counterparts (Terraneo, 2015; van Doorslaer et al., 2006). However, while a study found that gatekeeping could reduce inequalities in specialist care access (Reibling & Wendt, 2011), our results did not reveal such effect in cancer screening uptake inequalities.

As public health expenditure (PHE) increased, educational inequalities in cancer screening decreased, both in Pap smear and mammography, while higher OOPP revealed wider screening inequalities. These results are in line with studies which showed that more OOPP contributes to increasing healthcare foregoing (Bremer, 2014; Guessous et al., 2012; Mielck et al., 2009). They stress the impact of health service affordability among disadvantaged populations which became increasingly relevant in the backdrop of increasing unmet medical needs following the 2009 European economic crisis and subsequent governments' healthcare spending reduction (Burstrom, 2015; Reeves et al., 2015). Conversely, through healthcare cost redistribution in the population, higher PHE has the potential to reduce inequalities in preventive health services use, as our results showed, presumably through the reduced importance of private actors in the healthcare system and reduced individual's vulnerability to user-fees and costs. We may stress here the role of individual income in healthcare access as lower income populations are the most easily deterred by healthcare costs and face higher unmet medical needs (Mielck et al., 2007). Education level, our proxy for socioeconomic status, captures this economic barrier to screening, as the lower educated were shown to attend screening less than the higher educated in our results.

Concerning the moderating effect of specific social policies, countries with higher expenditure on sickness/healthcare had smaller screening inequalities between women of all education levels in both mammography and Pap smear. Disability and social exclusion expenditures had a similar effect and reduced disparities between the lower and higher educated groups. The events of sickness and disability more strongly affect disadvantaged groups since these involve expenses for households (and caregivers) which may entail financial strain, as well as social isolation and inactivity (Sjoberg, 2017). Moreover, women were shown to rely on sickness benefits more than men due to their more precarious work and work contracts, which are associated with part-time work and lower income, as well as their higher domestic workload (Spasova et al., 2016). Studies also revealed that women with disability are less likely to undergo mammography and Pap smear, especially those with lower social status (Ramjan et al., 2016). Consequently, social benefits can be crucial for more vulnerable groups to cope with the adverse economic conditions often associate with sickness and disability. The income redistribution these operate may not directly act upon preventive healthcare uptake but improve the living conditions of more disadvantaged women. This may in turn favour their contact with the healthcare system and make them more likely to receive information on “healthy lifestyle” and preventive health, while the higher educated are less in need of such support to access preventive healthcare since they are exposed to information on health through other sources (Gesthuizen et al., 2012). Hence, the lower educated who face cumulative disadvantages (of sickness, disability and lower socio-economic status) might benefit more from such social policies than the higher educated since they have less individual resources and depend more on collective resources. Social protection policies which transfer resources to specific (disadvantaged) groups may thus complement healthcare policies and help mitigate healthcare uptake obstacles (Israel, 2016). These not only lower income constraints, but also support the more disadvantaged groups to remain socially active, allowing them to afford social activities and meet people (Visser et al., 2018), and increase the resources needed to make informed choices and take effective actions towards health goals (Terraneo, 2015). Our results are in line with a population-level approach of prevention which attends to upstream socioeconomic determinants of health, as opposed to downstream approaches which focus on individual agency (McGill et al., 2015). They are also in line with a vulnerable populations perspective according to which vulnerable groups share underlying social conditions and characteristics and, thus, are exposed to multiple risk factors and are at “higher risk of risks” than other groups (Frohlich & Potvin, 2008). Consequently, vulnerable groups might derive more benefits from specific social policies than those groups who face lower risks.

Our results on the social exclusion expenditure moderating effect should be interpreted carefully since the Eurostat included countries’ expenditure “not elsewhere classified” in this category. Nonetheless, the sign of the interaction term pointed towards a reduction of screening uptake inequalities in contexts of more generous social spending. Concerning mammography, we found that countries with higher family expenditure had smaller screening inequalities. While Pap smear is performed during a GP visit or a routine gynaecological check-up, mammography involves referral to a specialist and thus two doctor appointments. Although this may vary through contexts, this increases its opportunity cost compared to a Pap smear, particularly for lower socio-economic status women, and family welfare provision might contribute to reducing such screening participation barrier.

The macro-level factors which moderated screening inequalities were not associated with countries’ screening participation levels (in Table 2), except for PC strength and expenditure. These factors may well contribute to reducing inequalities because the more disadvantaged groups benefit more from these, without increasing overall uptake levels. However, we should also point out that social spendings do not affect the entire population but, specifically, their beneficiary groups. These are only a part of the study sample which might not be large enough to reveal a statistical effect in the overall sample.

Our findings stress that policies and institutions affect the degree to which a woman's resources matter for preventive healthcare uptake. From a resource substitution perspective (Ross & Mirowsky, 2006), a favourable institutional context may provide the appropriate resources which women can substitute for the importance of their educational level in preventive healthcare use. This, in turn, can help mitigate the barriers to cancer screening uptake associated with a disadvantaged educational level (Willems et al., 2020). Drawing on institutional theories of health inequalities (Beckfield et al., 2015), we may advance that welfare institutions, through the (re)distribution of valued goods and resources, and through direct and indirect effects, shape the social determinants of preventive healthcare use.

Our study has some limitations. We did not fit full models to analyse the effects of multiple macro-level determinants on cancer screening participation due to the small number of countries in multilevel models. Part of the between-country variance remains unexplained by our models. Further macro-, meso- and micro-level determinants may differ across countries and could be examined in future research in order to explain the between-country variance left unexplained in the present study. We were not able to examine whether specific social spending areas were associated with cancer screening participation in their target groups due to limited (sub)sample size. Further research could examine the impact of specific social expenditures on the cancer screening participation of the groups they target. The indicators used in this study rely on national averages which conceal regional differences. Future analysis could benefit from a lower level of disaggregation, which was not possible with the EHIS data, particularly since many systems have shown a trend towards decentralised regulation and report geographic inequalities. Disaggregated social expenditure data should be interpreted carefully since some countries may use different types of benefits for similar objectives. Nevertheless, such data is used in comparative health research to analyse the effect of disaggregated social expenditure (de Breij et al., 2020) and has proved to meaningfully reflect patterns of welfare spending in Europe (Kuitto, 2011). Some limitations stemmed from the EHIS dataset. The cross-sectional nature of the data hinders causal inferences. Nevertheless, the literature provides evidence of a causal link between education level and health (Silles, 2009) and our outcomes occurred after the participants had completed their education. Recall and social desirability bias may affect self-reported screening data. Studies showed that women tend to over-report screening, however, self-reporting of cancer screening participation was shown to be moderately to highly accurate (Caplan et al., 2003; Rauscher et al., 2008). Age was provided in five-year groups and hence we were not able to adjust samples for countries whose screening age recommendations differed from the European guidelines. The EHIS did not collect information on women's cancer history nor on the reason for screening. Thus, we were not able to control for screening uptake in the context of treatment or previous diagnosis, or in case of symptoms. Opportunistic screening persists in every country alongside organised screening programmes, yet the data did not allow to control for a woman's cancer screening uptake through a programme or opportunistically. Nonetheless, our models controlled for women's health status and healthcare utilisation.

This study also has essential strengths. It is among the first to examine the role of both welfare and healthcare determinants in cancer screening participation and inequalities cross-nationally in both Pap smear and mammography. Screening participation levels and inequalities may have societal and economic consequences and contribute to reproducing health inequalities. Unlike cross-national studies which used aggregated indices to characterise countries, we included 14 macro-level determinants in order identify which determinants affected cancer screening uptake and educational inequalities. Healthcare features relevant to healthcare access and availability were selected and analysed. Social spending was disaggregated to examine the effects of specific types of social policy areas. This is particularly relevant since countries have different social protection expenditure priorities and a higher spending in one social policy area does not necessarily involve a higher spending in another area (Castles, 2009).

## 6 Conclusion

This study shows that social protection policies and healthcare system characteristics affect cancer screening uptake and educational differences in screening participation. These macro-level determinants interact with the individuals' resources and thus shape the extent to which an individual's education level (as a proxy for socioeconomic position) determines healthcare access. Favourable institutions and policies, through welfare resources' (re)distribution and decommodifying arrangements, may compensate for an individual's disadvantaged social position and enhance preventive healthcare participation. Our findings contribute to better contextualising the cross-national heterogeneity in cancer screening participation and inequalities, beyond the sole effect of cancer screening programmes. They highlight the advantage of examining specific social policy areas and shed light on (sub)groups who face cumulative disadvantages and may benefit from further social policy attention to increase their participation in preventive healthcare. Our results bring attention to the relevance of upstream prevention interventions which address structural (population-level) socioeconomic determinants. Such interventions should also take into account the underlying conditions of vulnerable groups if they aim at improving access to cancer screening for all, or they may have the unwitting effect of increasing screening inequalities. Cancer screening strategies should be conceived and implemented at the light of the healthcare system context which moderates service access and supply. As it was shown, a gatekeeping system is associated with reduced screening uptake and we suggested that it may contribute to limiting the overscreening phenomenon highlighted by previous research in Pap smear. Finally, a stronger primary care system, with a central role of the GP in first contact care and medical advocacy, may have the potential to reduce inequalities in cancer screening.

## CRediT authorship contribution statement

**Vladimir Jolidon:** Conceptualization, Methodology, Formal analysis, Writing – original draft, Writing – review & editing, Approved the final manuscript. **Piet Bracke:** Conceptualization, Writing – review & editing, Supervision, Approved the final manuscript, Funding acquisition, Project administration. **Claudine Burton-Jeangros:** Conceptualization, Writing – review & editing, Supervision, Approved the final manuscript, Funding acquisition, Project administration.

## Declaration of competing interest

None.
