# Supplementary material for: Macro-contextual determinants of cancer screening participation and inequalities: A multilevel analysis of 29 European countries
Source: SSM Popul Health. 2021 May 28;15:100830. doi: 10.1016/j.ssmph.2021.100830 (PMC8184663; doi:10.1016/j.ssmph.2021.100830)
Supplement: Multimedia component 1 [file mmc1.docx]

**Fig. S.1.** Level-2 (country) residuals (u0_j_) for Pap smear uptake multilevel model, with 95% confidence intervals. Romania was highlighted in red.

Note: Random intercept model adjusted for all level-1 (individual) confounders: education, age, cohabitation status, self-rated health, area of residence, work status, country of birth and visit to a GP in the past 12 months.

**Fig. S.2.** Level-2 (country) residuals (u0_j_) for mammography uptake multilevel model, with 95% confidence intervals. Romania was highlighted in red.

Note: Random intercept model adjusted for all level-1 (individual) confounders: education, age, cohabitation status, self-rated health, area of residence, work status, country of birth and visit to a GP in the past 12 months.

| **Table S.1** Comparison and harmonisation of variables in the European Health Interview Survey (EHIS) and the Swiss Health Interview Survey (SHIS) | | |
| --- | --- | --- |
| **Variable** | **EHIS** | **SHIS** |
| Pap smear uptake | Respondents were asked when they last had a Pap smear: ‘within the past 12 months, 1 to less than 2 years ago, 2 to less than 3 years ago, more than 3 years ago, or never’. We recoded as: had a Pap smear in the past 3 years, (0) no, (1) yes. | Respondents were asked for the date of their last Pap smear. The SHIS subtracts it from the interview date and provides the time since last smear test of the respondent. We recoded as: had a Pap smear in the past 3 years, (0) no, (1) yes. |
| Mammography uptake | Respondents were asked when they last had a mammography: ‘within the past 12 months, 1 to less than 2 years ago, 2 to less than 3 years ago, more than 3 years ago, or never’. We recoded as: had a mammography in the past 2 years, (0) no, (1) yes. | Respondents were asked for the date of their last mammography. The SHIS subtracts it from the interview date and provides the time since last mammography of the respondent. We recoded as: had a mammography in the past 2 years, (0) no, (1) yes. |
| Education | Followed ISCED classification | Followed ISCED classification |
| Age | 5-year age groups | Age (continuous), we recoded in 5-year age groups |
| Cohabitation status | Followed Eurostat “Core Social Variables”. | Followed Eurostat “Core Social Variables” |
| Self-rated health | Followed EHIS “Minimum European Health Module” | Followed EHIS “Minimum European Health Module” |
| Area of residence | Followed Eurostat “degree of urbanisation” based on LAUs (Local Administrative Units): ‘cities (densely populated areas), towns and suburbs (intermediate density areas), rural areas (thinly populated areas)’. We recoded as: (0) urban, (1) rural. | Followed Swiss Federal Statistical Office (OFS) “Swiss Geographic Levels” ^a^, which is harmonised with Eurostat’s LAUs (Local Administrative Units), provided as: (0) urban, (1) rural. |
| Work status | Followed Eurostat “Core Social Variables” | Followed Eurostat “Core Social Variables” |
| Country of birth | Followed Eurostat “Core Social Variables”: ‘native-born, born in another EU Member State, born in non-EU country’. We recoded as: (0) native, (1) born abroad. | Followed Swiss Federal Statistical Office (OFS) classification, provided as: (0) Swiss national, (1) Foreign national. |
| Last GP visit | Last visit to a general practitioner or family doctor: ‘less than 12 months ago, more than 12 months ago, never’. We recoded as: (0) GP visit less than 12 months ago, (1) more than 12 months ago. | Number of visits to a general practitioner or family doctor in the past 12 months. We recoded as: (0) GP visit less than 12 months ago, (1) more than 12 months ago. |
| ^a^ Swiss Federal Statistical Office (OFS). (2005). Recensement fédéral de la population 1990: Les niveaux géographiques de la Suisse. Neuchâtel: OFS.  *Sources:*  Eurostat. (2013). European Health Interview Survey (EHIS wave 2): Methodological manual. Luxembourg: Publications Office of the European Union.  Swiss Federal Statistical Office (OFS). (2019). Enquête suisse sur la santé 1992 à 2017: Documentation des indices de 1992 à 2017. Neuchâtel: OFS. | | |

| **Table S.2** Descriptive statistics for Pap smear (n= 99 715) and mammography (n=54 557) samples | | |
| --- | --- | --- |
|  | Pap smear in the past 3 years in women aged 25-64 | Mammography in the past 2 years in women aged 50-69 |
|  | N (%) | N (%) |
| Education |  |  |
| high | 34276 (34.4) | 12623 (23.1) |
| middle | 44279 (44.4) | 23827 (43.7) |
| low | 21160 (21.2) | 18107 (33.2) |
| Age |  |  |
| 25-29 | 8289 (8.3) |  |
| 30-34 | 10711 (10.7) |  |
| 35-39 | 12139 (12.2) |  |
| 40-44 | 13455 (13.5) |  |
| 45-49 | 14048 (14.1) |  |
| 50-54 | 14094 (14.1) | 14178 (26.0) |
| 55-59 | 13565 (13.6) | 13722 (25.2) |
| 60-64 | 13414 (13.5) | 13588 (24.9) |
| 65-69 | - | 13069 (24.0) |
| Cohabitation status |  |  |
| living in couple | 62128 (62.3) | 30389 (55.7) |
| living alone | 20805 (20.9) | 13377 (24.5) |
| other | 16782 (16.8) | 10791 (19.8) |
| Self-rated health |  |  |
| good | 72270 (72.5) | 31378 (57.5) |
| bad | 27445 (27.5) | 23179 (42.5) |
| Area of residence |  |  |
| urban | 65978 (66.2) | 35723 (65.5) |
| rural | 33737 (33.8) | 18834 (34.5) |
| Work status |  |  |
| employed | 65664 (65.9) | 23783 (43.6) |
| non-employed | 34051 (34.1) | 30774 (56.4) |
| Country of birth |  |  |
| native | 89070 (89.3) | 50326 (92.2) |
| abroad | 10645 (10.7) | 4231 (7.8) |
| Last GP visit |  |  |
| >12 months ago | 75281 (75.5) | 44158 (80.9) |
| <12 months ago | 24434 (24.5) | 10399 (19.1) |

| **Table S.3a** Macro-level factors per country: social protection expenditure, GDP per capita and unemployment rate | | | | | | | | | | |
| --- | --- | --- | --- | --- | --- | --- | --- | --- | --- | --- |
|  | Expenditure (% of GDP) | | | | | | | |  |  |
| Country (N=29) | Sickness/ healthcare | Disability | Old age | Survivors | Family/ children | Unemployment | Housing | Social exclusion | GPD per capita | Unemployment rate |
| Austria | 7.03 | 1.87 | 10.48 | 1.54 | 2.79 | 1.52 | 0.11 | 0.45 | 47922.0 | 5.10 |
| Belgium | 8.21 | 2.10 | 8.46 | 1.73 | 2.14 | 3.12 | 0.24 | 0.72 | 43671.1 | 8.30 |
| France | 8.90 | 2.00 | 11.41 | 1.62 | 2.48 | 1.81 | 0.84 | 0.91 | 39523.9 | 9.60 |
| Estonia | 4.05 | 1.76 | 6.33 | 0.07 | 1.58 | 0.43 | 0.04 | 0.10 | 27642.6 | 8.60 |
| Latvia | 3.28 | 1.20 | 7.21 | 0.21 | 1.17 | 0.58 | 0.12 | 0.14 | 22692.9 | 11.90 |
| Spain | 6.35 | 1.83 | 8.72 | 2.37 | 1.32 | 3.15 | 0.10 | 0.24 | 32434.0 | 25.60 |
| Greece | 5.33 | 1.55 | 12.67 | 2.27 | 1.09 | 1.31 | 0.00 | 0.07 | 26097.9 | 27.30 |
| Cyprus | 3.69 | 0.71 | 8.99 | 1.28 | 1.47 | 2.60 | 0.36 | 1.08 | 30375.2 | 15.80 |
| Slovenia | 7.27 | 1.52 | 10.15 | 1.60 | 1.83 | 0.81 | 0.02 | 0.65 | 29973.7 | 10.20 |
| Poland | 4.08 | 1.36 | 7.61 | 1.59 | 1.36 | 0.28 | 0.06 | 0.14 | 24719.2 | 10.20 |
| Slovakia | 5.47 | 1.60 | 6.99 | 0.92 | 1.72 | 0.56 | 0.04 | 0.41 | 27967.2 | 13.90 |
| Czech Republic | 5.95 | 1.34 | 8.59 | 0.70 | 1.76 | 0.60 | 0.25 | 0.31 | 30485.7 | 6.80 |
| Hungary | 4.81 | 1.47 | 9.45 | 1.24 | 2.35 | 0.45 | 0.31 | 0.11 | 24498.2 | 10.00 |
| Bulgaria | 4.43 | 1.36 | 7.65 | 0.97 | 1.82 | 0.54 | 0.00 | 0.26 | 16571.0 | 12.70 |
| Germany | 9.11 | 2.07 | 7.74 | 1.66 | 3.10 | 1.11 | 0.59 | 0.17 | 44993.9 | 5.20 |
| Denmark | 6.09 | 3.44 | 8.88 | 1.24 | 3.49 | 1.46 | 0.69 | 1.12 | 46726.9 | 6.90 |
| Finland | 7.11 | 3.17 | 9.62 | 0.74 | 3.08 | 1.88 | 0.56 | 0.88 | 41493.1 | 7.50 |
| Croatia | 6.73 | 2.66 | 6.79 | 2.08 | 1.57 | 0.55 | 0.02 | 0.23 | 21769.3 | 16.60 |
| Ireland | 7.87 | 1.13 | 5.91 | 0.48 | 2.05 | 2.90 | 0.68 | 0.20 | 47897.3 | 13.30 |
| Iceland | 7.74 | 3.14 | 4.69 | 0.39 | 2.54 | 0.76 | 0.71 | 0.63 | 44157.6 | 4.80 |
| Italy | 6.65 | 1.64 | 11.64 | 2.28 | 1.17 | 1.48 | 0.03 | 0.19 | 36314.7 | 11.90 |
| Luxembourg | 5.52 | 2.35 | 5.64 | 1.63 | 3.54 | 1.24 | 0.31 | 0.48 | 95590.5 | 5.70 |
| Lithuania | 4.00 | 1.38 | 6.44 | 0.44 | 1.01 | 0.37 | 0.03 | 0.61 | 26680.0 | 11.90 |
| Netherlands | 9.22 | 1.45 | 7.67 | 0.77 | 0.94 | 1.01 | 0.37 | 1.36 | 49241.5 | 6.70 |
| Norway | 6.85 | 3.60 | 7.40 | 0.21 | 2.96 | 0.49 | 0.13 | 0.71 | 66961.3 | 3.00 |
| Portugal | 6.19 | 1.93 | 11.07 | 1.80 | 1.20 | 1.77 | 0.00 | 0.24 | 27936.0 | 16.50 |
| Sweden | 7.09 | 3.20 | 10.16 | 0.33 | 2.90 | 1.02 | 0.45 | 0.70 | 46138.5 | 7.10 |
| United Kingdom | 8.65 | 1.61 | 10.86 | 0.09 | 2.83 | 0.56 | 1.42 | 0.73 | 39971.0 | 6.70 |
| Switzerland | 7.30 | 1.90 | 8.23 | 1.03 | 1.32 | 0.69 | 0.27 | 0.54 | 60108.5 | 4.60 |

| **Table S.3b** Macro-level factors per country: healthcare system characteristics and cancer screening programmes | | | | | | | | | | |
| --- | --- | --- | --- | --- | --- | --- | --- | --- | --- | --- |
| Country (N=29) | Pap smear screening programme^a^ | Mammography screening programme^a^ | OOPP as % of THE | PHE as % of GDP | GPs per 100 000 pop. | Gynaecologists per 100 000 pop. | GP referral^b^ | GP referral & capitation^c^ | PCE as a % of GDP | PC strength^d^ |
| Austria | 2 | 1 | 19.17 | 7.61 | 164.20 | 20.85 | 0 | 0 | 3.30 | 2.24 |
| Belgium | 2 | 0 | 18.03 | 8.05 | 111.86 | 12.34 | 0 | 0 | 2.96 | 2.23 |
| France | 1 | 0 | 9.93 | 8.73 | 142.59 | 12.24 | 0 | 0 | 2.73 | 2.17 |
| Estonia | 0 | 0 | 22.61 | 4.54 | 79.82 | 22.84 | 1 | 1 | 1.84 | 2.30 |
| Latvia | 0 | 0 | 38.47 | 3.24 | 67.42 | 21.41 | 1 | 1 | 1.05 | 2.17 |
| Spain | 2 | 0 | 23.98 | 6.40 | 75.11 | 11.86 | 1 | 1 | 3.12 | 2.43 |
| Greece | 2 | 2 | 33.69 | 5.23 | 31.86 | 26.10 | 0 | 0 | 1.85 | 2.12 |
| Cyprus | 2 | 0 | 43.09 | 3.18 | 79.77 | 18.89 | 0 | 0 | 2.25 | 1.97 |
| Slovenia | 0 | 1 | 12.45 | 6.05 | 58.45 | 16.75 | 1 | 1 | 2.49 | 2.37 |
| Poland | 0 | 0 | 23.80 | 4.50 | 33.77 | 13.27 | 1 | 1 | 1.83 | 2.14 |
| Slovakia | 1 | 2 | 23.32 | 5.59 | 41.42 | 16.00 | 0 | 0 | 1.94 | 2.05 |
| Czech Republic | 1 | 0 | 13.59 | 6.53 | 70.10 | 25.32 | 0 | 0 | 2.22 | 2.16 |
| Hungary | 1 | 0 | 28.38 | 4.84 | 49.07 | 14.87 | 1 | 1 | 1.75 | 2.10 |
| Bulgaria | 2 | 2 | 47.10 | 4.07 | 65.81 | 19.38 | 1 | 1 | 1.08 | 2.14 |
| Germany | 2 | 0 | 13.28 | 9.16 | 97.47 | 24.82 | 0 | 0 | 3.30 | 2.22 |
| Denmark | 0 | 0 | 13.81 | 8.57 | 79.34 | 10.45 | 1 | 1 | 4.38 | 2.39 |
| Finland | 0 | 0 | 19.34 | 7.29 | 125.92 | 13.94 | 1 | 0 | 4.05 | 2.31 |
| Croatia | 1 | 0 | 8.83 | 5.53 | 78.39 | 17.89 | 1 | 1 | 1.72 | . |
| Ireland | 0 | 0 | 14.28 | 7.32 | 233.16 | 7.22 | 1 | 1 | 3.19 | 2.18 |
| Iceland | 0 | 0 | 18.26 | 6.80 | 58.07 | 13.59 | 0 | 0 | 2.41 | 1.84 |
| Italy | 1 | 0 | 21.76 | 6.81 | 88.86 | 19.67 | 1 | 1 | 2.08 | 2.34 |
| Luxembourg | 2 | 0 | 11.07 | 4.73 | 85.95 | 15.83 | 0 | 0 | 1.73 | 1.94 |
| Lithuania | 1 | 0 | 32.82 | 4.07 | 91.59 | 25.15 | 1 | 1 | 1.72 | 2.28 |
| Netherlands | 0 | 0 | 11.53 | 8.59 | 145.78 | 8.70 | 1 | 1 | 3.10 | 2.49 |
| Norway | 0 | 0 | 14.59 | 7.58 | 87.35 | 10.75 | 1 | 1 | 2.96 | 2.27 |
| Portugal | 1 | 1 | 26.96 | 6.08 | 217.49 | 16.04 | 1 | 0 | 3.66 | 2.41 |
| Sweden | 0 | 0 | 15.54 | 9.25 | 64.59 | 14.09 | 1 | 0 | 4.12 | 2.25 |
| United Kingdom | 0 | 0 | 15.04 | 7.75 | 77.77 | 11.45 | 1 | 1 | 3.26 | 2.51 |
| Switzerland | 2 | 1 | 28.35 | 7.25 | 105.61 | 19.79 | 0 | 0 | 3.49 | 2.05 |
| OOPP = out-of-pocket payments, THE = total health expenditure, PHE = public health expenditure, PCE = primary care expenditure, PC = primary care | | | | | | | | | | |
| ^a^ (0) organised nationwide programme, (1) ‘partial’ programme (pilot/rollout ongoing/regional programmes), (2) no programme | | | | | | | | | |  |
| ^b^ (0) no GP referral, (1) GP referral | | |  |  |  |  |  |  |  |  |
| ^c^ (0) no GP referral, (1) GP referral and GP paid by capitation | | | |  |  |  |  |  |  |  |
| ^b^ Croatia indicator was not available | |  |  |  |  |  |  |  |  |  |

| **Table S.4** Definitions of the functions of Eurostat social protection expenditures | |
| --- | --- |
| **Function** | **Brief description** |
| 1. Sickness/Healthcare | Income maintenance and support in cash in connection with physical or mental illness, excluding disability. Health care intended to maintain, restore or improve the health of the people protected irrespective of the origin of the disorder. |
| 1. Disability | Income maintenance and support in cash or kind (except health care) in connection with the inability of physically or mentally disabled people to engage in economic and social activities. |
| 1. Old age | Income maintenance and support in cash or kind (except health care) in connection with old age. |
| 1. Survivors | Income maintenance and support in cash or kind in connection with the death of a family member. |
| 1. Family/children | Support in cash or kind (except health care) in connection with the costs of pregnancy, childbirth and adoption, bringing up children and caring for other family members. |
| 1. Unemployment | Income maintenance and support in cash or kind in connection with unemployment. |
| 1. Housing | Help towards the cost of housing. |
| 1. Social exclusion | Benefits in cash or kind (except health care) specifically intended to where classified combat social exclusion where they are not covered by one of the other functions. |
| *Source:* Eurostat. 2019. European system of integrated social protection statistics - ESSPROS: Manual and user guideline. Luxembourg: Eurostat. | |

| **Table S.5** Multilevel models with association of primary care expenditure (PCE) with Pap smear and mammography uptake | | | | | | |
| --- | --- | --- | --- | --- | --- | --- |
|  | Model 2 | | | | | |
|  | Pap Smear (n= 99715) | | | Mammography (n= 54 557) | | |
|  | OR (SE) | MOR | VPC | OR (SE) | MOR | VPC |
| PCE % GDP | 0.948 (0.114) | 1.547 | 0.060 | 1.603** (0.241) | 1.683 | 0.083 |
| *p value ≤ 0.05, **p value ≤ 0.01, ***p value ≤ 0.001. | | | | | | |
| PCE = primary care expenditure, MOR = median odds ratio, VPC = variance partitioning coefficient | | | | | | |
| Note: Random intercept models adjusted for education, age, cohabitation status, self-rated health, area of residence, work status, country of birth, visit to a GP in the past 12 months, cancer screening programme and GDP per capita. | | | | | | |

| **Table S.6** Cross-level interaction between education and primary care expenditure (PCE) in Pap smear and mammography | | | | |
| --- | --- | --- | --- | --- |
|  | Model 3 | | | |
|  | Macro-level variable | | Education * Macro-level variable | |
|  | Pap Smear | Mammography | Pap Smear | Mammography |
|  | OR (SE) | OR (SE) | OR (SE) | OR (SE) |
| PCE % GDP * middle edu. | 0.888 (0.117) | 1.293 (0.202) | 1.092* (0.045) | 1.214*** (0.043) |
| PCE % GDP * low edu. |  |  | 1.253** (0.089) | 1.351*** (0.084) |
| *p value ≤ 0.05, **p value ≤ 0.01, ***p value ≤ 0.001. | | | | |
| Pap smear n= 99 715, Mammography n= 54 557 | | | | |
| PCE = primary care expenditure | | | | |
| Note: Random coefficients where included for education. Models were adjusted for age, cohabitation status, self-rated health, area of residence, work status, country of birth, visit to a GP in the past 12 months, cancer screening programme and GDP per capita. | | | | |

Low education Middle education High education

**Figure S.3** Predicted probabilities of Pap smear and mammography uptake by education levels and primary care expenditure

Low education Middle education High education

**Figure S.4** Predicted probabilities of Pap smear and mammography uptake by education levels and primary care strength
